# The Relationship Between Self-Rated Health and Use of Parks and Participation in Recreation Programs, United States, 1991 and 2015

**DOI:** 10.5888/pcd14.160441

**Published:** 2017-01-05

**Authors:** Nicholas A. D. Pitas, Austin G. Barrett, Andrew J. Mowen, Alan R. Graefe, Geoffrey C. Godbey, Christopher N. Sciamanna

**Affiliations:** 1 The Pennsylvania State University, Department of Recreation, Park, and Tourism Management, University Park, Pennsylvania; 2 The Pennsylvania State University, Milton S. Hershey Medical Center, Hershey, Pennsylvania.

## Abstract

We examined the relationship between self-rated health and use of parks and recreation program participation by using logistic regression to analyze data from representative national surveys conducted in 1991 and 2015. Neither park use nor program participation were significantly related to self-rated health in 1991; however, both were significantly related in 2015. The growing relationship between use of parks and recreation programs and self-rated health during this period is likely the result of broad national health promotion efforts and provides support for funding of capital and operational expenses for park and recreation services.

## Objectives

Public parks and organized recreation programs are assets for improving community health (1,2). Although many studies have examined the relationships between parks and physical activity, no national-level study has examined the relationship between local park use and participation in recreation programs in the context of health outcomes (1,2). Furthermore, no study has examined whether the relationship between park use, participation in recreation programs, and self-rated health has strengthened over time concurrent with national efforts to promote healthy living (3). To address these information gaps, we examined associations between the variables of park use, participation in recreation programs, and self-rated health in the United States in 1991 and 2015 while controlling for demographic characteristics of users and park proximity characteristics.

## Methods

Two cross-sectional telephone surveys were conducted by The Pennsylvania State University using a sample provided by Survey Sampling International (https://www.surveysampling.com/) to assess respondents’ use of local parks and their participation in organized recreation programs. Surveys were conducted in 1991 (1,305 respondents), and 2015 (1,144 respondents); both surveys consisted of the same questions and used the same data collection procedures. Surveys were administered to a nationally representative sample of the US population; names were purchased from Survey Sampling International. Self-rated health, the dependent variable, was assessed through the Short Form Health Survey, question 1 (SF-1) (www.rand.org/health/surveys_tools/mos/36-item-short-form.html), which asks, “In general, compared to other persons your age, would you say your health is excellent, very good, good, fair, poor?” The SF-1 is a robust predictor of illness and death and is valid as a single item (4,5). Responses were dichotomized as low or high, on the basis of whether scores were less than or equal to the median value or above the median value (6,7). Park use and participation in organized recreation programs were assessed as independent variables. Park use was measured as how often respondents used their local park areas: not at all or occasionally, or frequently (dichotomized to not at all/occasionally or frequently). Participation during the past 12 months in programs or activities organized by local park and recreation departments or at areas or facilities managed by a local park and recreation department was measured as yes or no. Sex, age (dichotomized as 18–64 y or ≥65), race (dichotomized as white or nonwhite), education (dichotomized to less than a college degree [high school diploma/general equivalency development or less, or completed some college or technical or vocational school] or college or more [undergraduate degree but no graduate degree or had completed a graduate degree]) were reported. Perceived proximity was assessed by asking whether a park, playground, or open space was within walking distance of the respondent’s home (yes or no).

We used logistic regression to test the relationship between self-rated health and park use and program participation. Demographic characteristics and perceived walking distance to a park were controlled for in each year’s model, which consisted of participants with complete data for all dependent and independent variables included (1,225 in 1991; 1,071 in 2015).

## Results

The survey response rate was 34.6% in 1991 and 38.0% in 2015. In both 1991 and 2015, most respondents in the sample were female (55.8% in 1991; 52.6% in 2015), aged 18 to 64 years (85.8% and 81.5%), and white (86.7% and 74.7%) (Table 1). Twenty-nine percent reported having a college education or more in 1991 compared with 43.0% in 2015; 71.5% perceived that they lived within walking distance of a park in 1991 compared with 67.8% in 2015. In 1991, 59.6% reported high self-rated health compared with 54.7% in 2015. 

**Table 1:** Demographic Characteristics of Participants and Park and Recreation Use in Two Cross-Sectional Telephone Surveys Assessing Self-Rated Health and Use of Local Parks and Recreation Programs, United States, 1991 and 2015^a^

Characteristic	1991 Sample (N = 1,305), %	2015 Sample (N = 1,144), %
**Self-rated health^b^**		
Low	40.4	45.3
High	59.6	54.7
**Park use**		
Not at all/occasionally	75.9	74.5
Frequently	24.1	25.5
**Program participation**		
No	69.9	68.1
Yes	30.1	31.9
**Sex**		
Female	55.8	52.6
Male	44.2	47.4
**Age^c^, y**		
18–64	85.8	81.5
≥65	14.2	18.5
**Race^d^**		
Nonwhite^e^	13.3	25.3
White	86.7	74.7
**Education^f^**		
Less than college graduate	70.7	57.0
College graduate or greater	29.3	43.0
**Perceived proximity to parks^g^**		
Within walking distance	71.5	67.8
Not within walking distance	28.5	32.2

a The response rate in 1991 was 34.6%, and the response rate in 2015 was 38.0%.

b *P* = .02.

c *P* = .006.

d *P* < .001.

e Respondents who identified as Hispanic, non-Hispanic black, Asian or Pacific Islander, or American Indian or Alaska Native.

f “Less than college graduate” comprised respondents who had a high school diploma/GED or less, or completed some college or technical or vocational school. “College graduate or more” comprised respondents who had an undergraduate degree but no graduate degree or had completed a graduate degree.

g *P* = .049.

In 1991, neither park use nor program participation were significantly related to self-rated health (Table 2). Of the remaining variables, only education was significantly associated with self-rated health; respondents with a college degree or more were more likely to report high self-rated health in 1991 (adjusted odds ratio [AOR] = 1.54; 95% confidence interval [CI], 1.18–2.00) than those with less than a college degree.

**Table 2:** Association Between Use of Parks and Recreation Programs and High Self-Rated Health Among Participants in Two Cross-Sectional Telephone Surveys Assessing Use of Local Parks and Recreation Programs, United States,1991 and 2015

Variable	High^a^ Self-Rated Health	
	1991 Sample (N = 1,225)	2015 Sample (N = 1,071)
	Adjusted Odds Ratio (95% Confidence Interval)	
**Park use^b^**		
Not at all/occasionally	1 [Reference]	
Frequently	1.06 (0.81–1.40)	1.36 (1.01–1.83)^c^
**Program participation^d^**		
No	1 [Reference]	
Yes	1.19 (0.91–1.54)	1.60 (1.21–2.12)^e^
**Sex**		
Female	1 [Reference]	
Male	0.97 (0.77–1.23)	0.99 (0.77–1.28)
**Age, y**		
18–64	1 [Reference]	
≥65	0.76 (0.54–1.05)	1.11 (0.80–1.54)
**Race**		
Nonwhite^f^	1 [Reference]	
White	1.16 (0.83–1.63)	1.17 (0.88–1.57)
**Education^g^**		
Less than college graduate	1 [Reference]	
College graduate or more	1.54 (1.18–2.00)^h^	1.77 (1.36–2.29)^i^
**Perceived proximity to park**		
Within walking distance	1 [Reference]	
Not within walking distance	0.92 (0.71–1.19)	1.10 (0.84–1.44)

a High self-rated health included all responses that were greater than the median value for the question, “In general, compared to other persons your age, would you say your health is excellent, very good, good, fair, poor?” Demographic characteristics and perceived walking distance to a park were controlled for in each year’s model.

b How often a respondent used local park areas.

c *P* = .046.

d Whether or not a respondent participated in organized recreation programs or activities that were sponsored by or took place in areas or facilities managed by their local government’s recreation and parks department within the past 12 months.

e *P *= .001.

f Respondents who identified as Hispanic, non-Hispanic black, Asian or Pacific Islander, or American Indian or Alaska Native.

g “Less than college graduate” comprised respondents who had a high school diploma or general educational development or less, or completed some college or technical or vocational school. “College graduate or more” comprised respondents who had an undergraduate degree but no graduate degree or had completed a graduate degree.

h *P* = .001.

i *P* <.001.

In 2015, after controlling for respondents’ demographic characteristics and perceived proximity to a park, frequent park users were more likely to report high self-rated health than nonfrequent park users (AOR = 1.36; 95% CI, 1.01–1.83) (Table 2). Likewise, in 2015, program participants were more likely to report high self-rated health than nonprogram participants (AOR = 1.60; 95% CI, 1.21–2.12). As in 1991, education remained significantly related to self-rated health in 2015; respondents who achieved a college degree or more were more likely to report high self-rated health (AOR = 1.77; 95% CI, 1.36–2.29) than those without a college degree. 

## Discussion

Local park and recreation services can improve public health (1,2,8). Parks provide access to natural areas, which may be limited for residents of suburban or urban areas, and provide physiological, social, and mental benefits (9). Research suggests a positive relationship between park use and physical activity (1,2,8) and between program participation and physical activity (10,11), although we know less about the role of parks and organized recreation programs in relation to overall health. By using national data, we found an increasing association over time between park use, program participation, and self-rated health. This study supports the importance of locally offered recreation facilities and programs to contribute to residents’ health and provides evidence to support adequate funding for both quality recreation facilities and programming as part of the public health infrastructure.

Study results suggest that the effect of park use and participation in recreational programs on health has strengthened in the United States during the last 2 decades. Whereas these 2 variables were not significantly related to self-rated health in 1991, by 2015 their importance had increased significantly. Considering this trend, adequate investment in parks and programs is increasingly important to combat nationwide health concerns. Although we found notable differences in several demographic characteristics among survey respondents, their relationship to self-rated health was consistent across time, and their effects were controlled for in both years’ models.

Limitations of our study are its cross-sectional nature, which makes it impossible to infer causality. In addition, analyses relied on self-reported data for park use and program participation. Future national studies of parks should consider a wider range of health metrics and a longitudinal design. Such evidence would allow advocates to argue more effectively for funding and for more effective design and implementation of both parks and organized recreation services.

## References

[1] Cohen DA , McKenzie TL , Sehgal A , Williamson S , Golinelli D , Lurie N . Contribution of public parks to physical activity. Am J Public Health 2007;97(3):509–14. 10.2105/AJPH.2005.072447 17267728 PMC1805017

[2] Kaczynski AT , Henderson KA . Environmental correlates of physical activity: a review of evidence about parks and recreation. Leis Sci 2007;29(4):315–54. 10.1080/01490400701394865

[3] Pate RR . A national physical activity plan for the United States. J Phys Act Health 2009;6(6, Suppl 2):S157–8. 10.1123/jpah.6.s2.s157 20120124

[4] Latham K , Peek CW . Self-rated health and morbidity onset among late midlife US adults. J Gerontol B Psychol Sci Soc Sci 2013;68(1):107–16. 10.1093/geronb/gbs104 23197340 PMC3605944

[5] DeSalvo KB , Bloser N , Reynolds K , He J , Muntner P . Mortality prediction with a single general self-rated health question. A meta-analysis. J Gen Intern Med 2006;21(3):267–75. 10.1111/j.1525-1497.2005.00291.x 16336622 PMC1828094

[6] Manor O , Matthews S , Power C . Dichotomous or categorical response? Analysing self-rated health and lifetime social class. Int J Epidemiol 2000;29(1):149–57. 10.1093/ije/29.1.149 10750617

[7] DeSalvo KB , Fan VS , McDonell MB , Fihn SD . Predicting mortality and healthcare utilization with a single question. Health Serv Res 2005;40(4):1234–46. 10.1111/j.1475-6773.2005.00404.x 16033502 PMC1361190

[8] Bedimo-Rung AL , Mowen AJ , Cohen DA . The significance of parks to physical activity and public health: a conceptual model. Am J Prev Med 2005;28(2, Suppl 2):159–68. 10.1016/j.amepre.2004.10.024 15694524

[9] Maller C , Townsend M , St Leger L , Henderson-Wilson C , Pryor A , Prosser L , Healthy parks healthy people: the health benefits of contact with nature in a park context. George Wright Forum 2009;26(2):51–83.

[10] Cohen DA , Sehgal A , Williamson S , Marsh T , Golinelli D , McKenzie TL . New recreational facilities for the young and the old in Los Angeles: policy and programming implications. J Public Health Policy 2009;30(1, Suppl 1):S248–63. 10.1057/jphp.2008.45 19190577 PMC2764332

[11] Cohen DA , Han B , Nagel CJ , Harnik P , McKenzie TL , Evenson KR , The first national study of neighborhood parks: implications for physical activity. Am J Prev Med 2016;51(4):419–26. 10.1016/j.amepre.2016.03.021 27209496 PMC5030121

