# Erratum, Vol. 14, January 5 Release

**DOI:** 10.5888/pcd14.160441e

**Published:** 2017-01-26

In the article “The Relationship Between Self-Rated Health and Use of Parks and Participation in Recreation Programs, United States, 1991 and 2015,” the author reported an incorrect value in the last row, last cell of Table 2. That value should be 1.10 (0.84–1.44), not 1.10 (8.38–1.44). The table was corrected on January 12, 2017, and the revised article appears online at www.cdc.gov/pcd/issues/2017/16_0441.htm. We regret any confusion this incorrect value may have caused.

